# Computational Fact Checking from Knowledge Networks

**DOI:** 10.1371/journal.pone.0128193

**Published:** 2015-06-17

**Authors:** Giovanni Luca Ciampaglia, Prashant Shiralkar, Luis M. Rocha, Johan Bollen, Filippo Menczer, Alessandro Flammini

**Affiliations:** 1 Center for Complex Networks and Systems Research, Indiana University, Bloomington, Indiana, United States of America; 2 Instituto Gulbenkian de Ciencia, Oeiras, Portugal; Centre de Physique Théorique, FRANCE

## Abstract

Traditional fact checking by expert journalists cannot keep up with the enormous volume of information that is now generated online. Computational fact checking may significantly enhance our ability to evaluate the veracity of dubious information. Here we show that the complexities of human fact checking can be approximated quite well by finding the shortest path between concept nodes under properly defined semantic proximity metrics on knowledge graphs. Framed as a network problem this approach is feasible with efficient computational techniques. We evaluate this approach by examining tens of thousands of claims related to history, entertainment, geography, and biographical information using a public knowledge graph extracted from Wikipedia. Statements independently known to be true consistently receive higher support via our method than do false ones. These findings represent a significant step toward scalable computational fact-checking methods that may one day mitigate the spread of harmful misinformation.

## Introduction

Online communication platforms, in particular social media, have created a situation in which the proverbial lie “can travel the world before the truth can get its boots on.” Misinformation [1], astroturf [2], spam [3], and outright fraud [4] have become widespread. They are now seemingly unavoidable components of our online information ecology [5] that jeopardize our ability as a society to make rapid and informed decisions [6–10].

While attempts to partially automate the detection of various forms of misinformation are burgeoning [11–15], automated reasoning methods are hampered by the inherent ambiguity of language and by deliberate deception. However, under certain conditions, reliable knowledge transmission can take place online [16]. For example, Wikipedia, the crowd-sourced online encyclopedia, has been shown to be nearly as reliable as traditional encyclopedias, even though it covers many more topics [17]. It now serves as a large-scale knowledge repository for millions of individuals, who can also contribute to its content in an open way. Vandalism, bias, distortions, and outright lies are frequently repaired in a matter of minutes [18]. Its continuous editing process even indicates signs of collective human intelligence [19].

Here we show that we can leverage any collection of factual human knowledge, such as Wikipedia, for automatic fact checking [20]. Loosely inspired by the principle of epistemic closure [21], we computationally gauge the support for statements by mining the connectivity patterns on a knowledge graph. Our initial focus is on computing the support of simple statements of fact using a large-scale knowledge graph obtained from Wikipedia. More in general, fact checking can be seen as a special case of link prediction in knowledge graphs [22].

### Knowledge Graphs

Let a *statement of fact* be represented by a subject-predicate-object triple, e.g., (“Socrates,” “is a,” “person”). A set of such triples can be combined to produce a *knowledge graph* (KG), where nodes denote *entities* (i.e., subjects or objects of statements), and edges denote predicates. Given a set of statements that has been extracted from a knowledge repository—such as the aforementioned Wikipedia—the resulting KG network represents all factual relations among entities mentioned in those statements. Given a new statement, we expect it to be true if it exists as an edge of the KG, or if there is a short path linking its subject to its object within the KG. If, however, the statement is untrue, there should be neither edges nor short paths that connect subject and object.

In a KG distinct paths between the same subject and object typically provide different factual support for the statement those nodes represent, even if the paths contain the same number of intermediate nodes. For example, paths that contain generic entities, such as “United States” or “Male,” provide weaker support because these nodes link to many entities and thus yield little specific information. Conversely, paths comprised of very specific entities, such as “positronic flux capacitor” or “terminal deoxynucleotidyl transferase,” provide stronger support. A fundamental insight that underpins our approach is that the definition of path length used for fact checking should account for such information-theoretic considerations.

**Fig 1:**
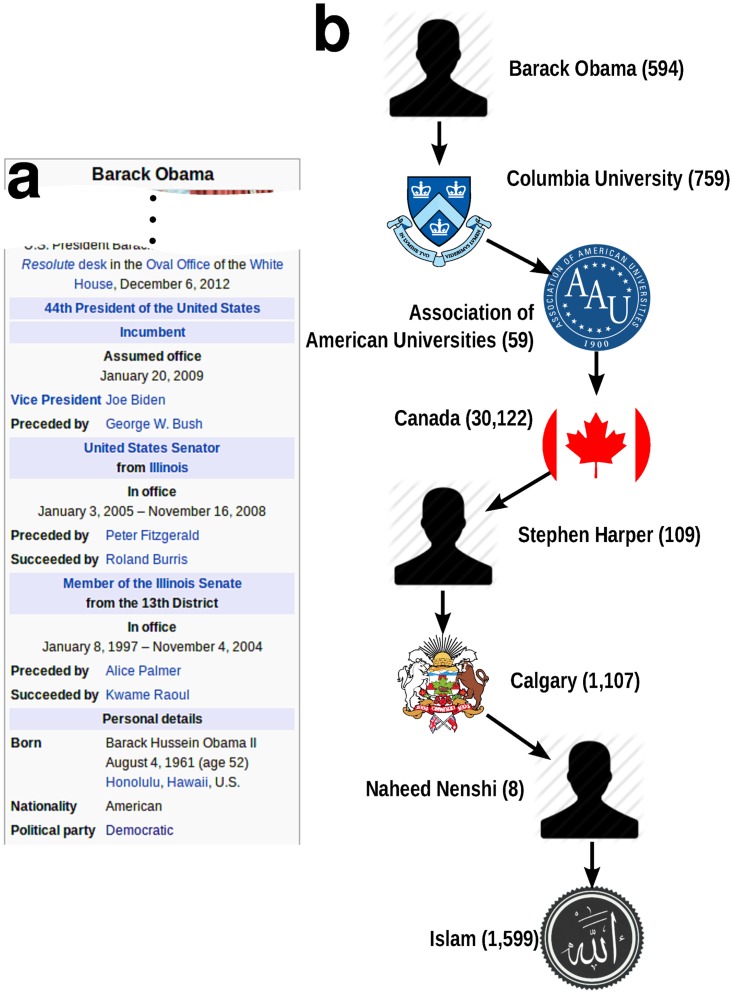
Using Wikipedia to fact-check statements. **(a)** To populate the knowledge graph with facts we use structured information contained in the ‘infoboxes’ of Wikipedia articles (in the figure, the infobox of the article about *Barack Obama*). **(b)** Using the Wikipedia Knowledge Graph, computing the truth value of a subject-predicate-object statement amounts to finding a path between subject and object entities. In the diagram we plot the shortest path returned by our method for the statement “*Barack Obama* is a *muslim*.” Numbers in parentheses indicate the degree of the nodes. The path traverses high-degree nodes representing generic entities, such as *Canada*, and is assigned a low truth value.

To test our method we use the DBpedia database [23], which consists of all factual statements extracted from Wikipedia’s “infoboxes” (see Fig 1(a)). From this data we build the large-scale *Wikipedia Knowledge Graph* (WKG), with 3 million entity nodes linked by approximately 23 million edges (see Materials and Methods). Since we use only facts within infoboxes, the WKG contains the most uncontroversial information available on Wikipedia. This conservative approach is employed to ensure that our process relies as much as possible on a human-annotated, collectively-vetted factual basis. The WKG could be augmented with automatic methods to infer facts from text and other unstructured sources available online. Indeed, other teams have proposed methods to infer knowledge from text [24] to be employed in large and sophisticated rule-based inference models [24–26]. Here we focus on the feasibility of automatic fact checking using *simple* network models that leverage DBpedia. For this initial goal, we do not need to enhance the WKG, but such improvements can later be incorporated.

### Semantic Proximity from Transitive Closure

Let the WKG be an undirected graph *G* = (*V*, *E*) where *V* is a set of concept nodes and *E* is a set of predicate edges (see Materials and Methods). Two nodes *v*, *w* ∈ *V* are said to be *adjacent* if there is an edge between them (*v*, *w*) ∈ *E*. They are said to be *connected* if there a sequence of *n* ≥ 2 nodes *v* = *v*_1_, *v*_2_, … *v*_*n*_ = *w*, such that, for *i* = 1, …, *n*−1 the nodes *v*_*i*_ and *v*_*i*+1_ are adjacent. The *transitive closure* of *G* is G* = (*V*, E*) where the set of edges is closed under adjacency, that is, two nodes are adjacent in G* *iff* they are connected in *G* via at least one path. This standard notion of closure has been extended to weighted graphs, allowing adjacency to be generalized by measures of path length [27], such as the semantic proximity for the WKG we introduce next.

The truth value *τ*(*e*) ∈ [0, 1] of a new statement *e* = (*s*, *p*, *o*) is derived from a transitive closure of the WKG. More specifically, the truth value is obtained via a path evaluation function: *τ*(*e*) = max 𝒲(*P*_*s*,*o*_). This function maps the set of possible paths connecting *s* and *o* to a truth value *τ*. A path has the form *P*_*s*,*o*_ = *v*_1_ *v*_2_…*v*_*n*_, where *v*_*i*_ is an entity node, (*v*_*i*_, *v*_*i*+1_) is an edge, *n* is the path length measured by the number of its constituent nodes, *v*_1_ = *s*, and *v*_*n*_ = *o*. Various characteristics of a path can be taken as evidence in support of the truth value of *e*. Here we use the *generality* of the entities along a path as a measure of its length, which is in turn aggregated to define a *semantic proximity*:

$$\begin{array}{c} 𝒲\left(P_{s,o}\right)=𝒲\left(v_{1}\ldots v_{n}\right)={\left[1+\sum_{i=2}^{n-1}\log k(v_{i})\right]}^{-1} \end{array}$$ (1)

where *k*(*v*) is the degree of entity *v*, i.e., the number of WKG statements in which it participates; it therefore measures the generality of an entity. If *e* is already present in the WKG (i.e., there is an edge between *s* and *o*), it should obviously be assigned maximum truth. In fact 𝒲 = 1 when *n* = 2 because there are no intermediate nodes. Otherwise an indirect path of length *n* > 2 may be found via other nodes. The truth value *τ*(*e*) maximizes the semantic proximity defined by Eq 1, which is equivalent to finding the shortest path between *s* and *o* [27], or the one that provides the maximum information content [28, 29] in the WKG. The transitive closure of weighted graphs equivalent to finding the shortest paths between every pair of nodes is also known as the *metric closure* [27]. This approach is also related to the Path Ranking Algorithm [30], except that here we use the shortest path (equivalent to maximum probability) rather than combining a sample of bounded-length paths in a learning framework.

Fig 1(b) depicts an example of a shortest path on the WKG for a statement that yields a low truth value. Note that in this specific formulation we disregard the semantics of the predicate, therefore we are only able to check statements with the simplest predicates, such as “is a”; negation, for instance, would require a more sophisticated definition of path length.

Alternative definitions of *τ*(*e*) are of course possible. Instead of shortest paths, one could use a different optimization principle, such as widest bottleneck, also known as the *ultra-metric closure* [27], which corresponds to maximizing the path evaluation function 𝒲_*u*_:

$$\begin{array}{c} {𝒲}_{u}\left(P_{s,o}\right)=\;{𝒲}_{u}(v_{1}\ldots v_{n})=\{\begin{array}{cc} 1 & n=2\\ {\left[1+\max_{i=2}^{n-1}\{\log k(v_{i})\}\right]}^{-1} & n>2. \end{array} \end{array}$$ (2)

Or it could be possible to retain the original directionality of edges and have a directed WKG instead of an undirected one. As described next, we evaluated alternative definitions of *τ*(*e*) and found Eq 1 to perform best.

## Results

### Calibration

**Fig 2:**
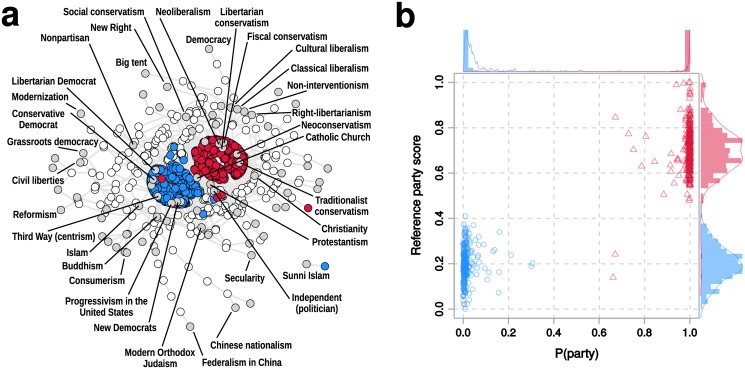
Ideological classification of the US Congress based on truth values. **(a)** Ideological network of the 112th US Congress. The plot shows a subset of the WKG constituted by paths between Democratic or Republican members of the 112th US Congress and various ideologies. Red and blue nodes correspond to members of Congress, gray nodes to ideologies, and white nodes to vertices of any other type. The position of the nodes is computed using a force-directed layout [33], which minimizes the distance between nodes connected by an edge weighted by a higher truth value. For clarity only the most significant paths, whose values rank in the top 1% of truth values, are shown. **(b)** Ideological classification of members of the 112th US Congress. The plot shows on the *x* axis the party label probability given by a Random Forest classification model trained on the truth values computed on the WKG, and on the *y* axis the reference score provided by dw-nominate. Red triangles are members of Congress affiliated to the Republican party and blue circles to the Democratic party. Histograms and density estimates of the two marginal distributions, color-coded by actual affiliation, are shown on the top and right axes.

Our fact-checking method requires that we define a measure of path semantic proximity by selecting a transitive closure algorithm (the shortest paths of Eq 1 or the widest bottleneck paths of Eq 2) and a directed or undirected WKG representation. To evaluate these four combinations empirically, let us attempt to infer the party affiliation of US Congress members. In other words, we want to compute the support of statements like “*x* is a member of *y*” where *x* is a member of Congress and *y* is a political party. We consider all members of the 112th US Congress that are affiliated with either the Democratic or Republican party (Senate: *N* = 100; House: *N* = 445). We characterize each member of Congress with its semantic proximity to all nodes in the WKG that represent ideologies. This yields an *N* × *M* feature matrix ℱ_tc_ for each of the four transitive closure methods. Panel (a) of Fig 2 illustrates the proximity network obtained from ℱ_tc_ that connects members of the 112th Congress and their closest ideologies, as computed using Eq 1. A high degree of ideological polarization can be observed in the WKG, consistent with blogs [31] and social media [32].

We feed ℱ_tc_ into off-the-shelf classifiers (see Materials and Methods). As shown in Table 1, the metric closure on the undirected graph gives the most accurate results. Therefore, we continue to use this combination in our semantic proximity computations when performing the validation tasks described below.

**Table 1:** Transitive closure calibration.

		Directed		Undirected	
		k-NN	RF	k-NN	RF
House	Metric	96	99	97	99
House	Ultra-metric	56	57	53	57
Senate	Metric	70	100	96	100
Senate	Ultra-metric	49	39	70	61

Area under Receiver Operating Characteristic (ROC) curve of two classifiers, random forests (RF) and *k*-nearest neighbors (*k*-NN) on the ideological classification task.

To evaluate the overall performance of the calibrated model, we also compared it against dw-nominate, the state of the art in political classification [34]. This model is not based on data from a knowledge graph, but on explicit information about roll-call voting patterns. Comparing our classification results with such a baseline is also useful to gauge the quality of the latent information contained in the WKG for the task of political classification. As shown in panel (b) of Fig 2, a Random Forests classifier trained on our truth values matches the performance of dw-nominate.

### Value of indirect connections

Most of the WKG information that our fact checker exploits is provided by indirect paths (i.e., comprising *n* > 2 nodes). To demonstrate this, we compare the calibrated model of Eq 1 to the fact checker’s performance with only the information in the infoboxes.

In practice, we compute an additional feature matrix ℱ_b_, using the same sequence of steps outlined in the calibration phase, but additionally constraining the shortest path algorithm to use only paths (if any) with exactly *n* = 2 nodes, i.e., direct edges. Thus ℱ_b_ encodes only the information of the infoboxes of the politicians. The results from 10-fold cross validation using ℱ_tc_ and ℱ_b_ are shown in Table 2. The same off-the-shelf classifiers, this time trained on ℱ_b_, perform only slightly better than random, thus confirming that the truth signal is yielded by the structure of indirect connections in the WKG.

**Table 2:** Ideological classification results.

	RF		*k*-NN	
Dataset	F-score	AUROC	F-score	AUROC
Transitive closure ℱ_tc_				
Senate	0.99	1.00	0.91	0.96
House	0.99	1.00	0.90	0.97
Infoboxes ℱ_b_				
Senate	0.66	0.46	0.62	0.54
House	0.54	0.66	0.68	0.54

Out-of-sample F-score and Area Under Receiver Operating Characteristic (AUROC) of random forest (RF) and *k*-nearest neighbors (*k*-NN) classifiers trained on truth scores computed by the fact checker, using either the transitive closure or solely information from infoboxes.

### Validation on factual statements

**Fig 3:**
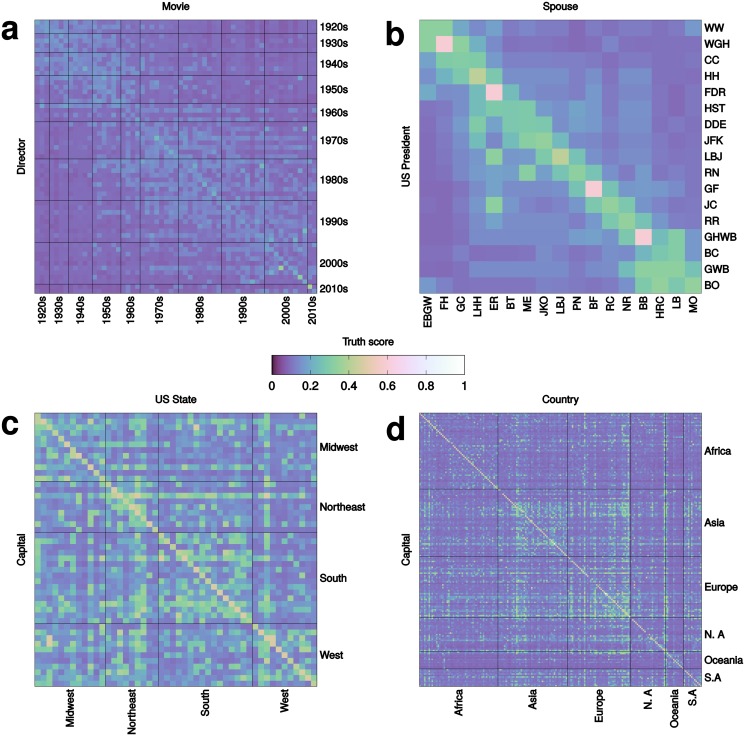
Automatic truth assessments for simple factual statements. In each confusion matrix, rows represent subjects and columns represent objects. The diagonals represent true statements. Higher truth values are mapped to colors of increasing intensity. **(a)** Films winning the Oscar for Best Movie and their directors, grouped by decade of award (see the complete list in the S1 Text). **(b)** US presidents and their spouses, denoted by initials. **(c)** US states and their capitals, grouped by US Census Bureau-designated regions. **(d)** World countries and their capitals, grouped by continent.

**Fig 4:**
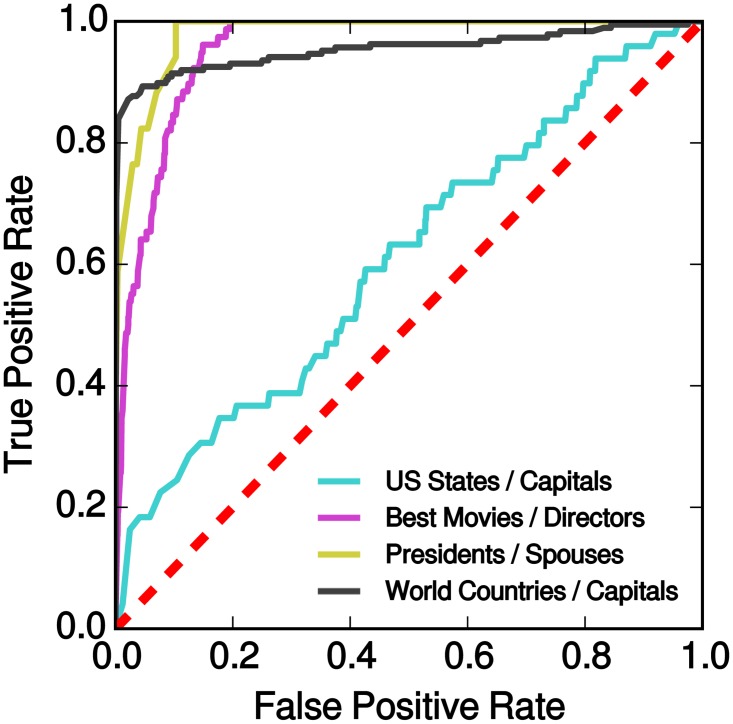
Receiver Operating Characteristic for the multiple questions task. For each confusion matrix depicted in Fig 3 we compute ROC curves where true statements correspond to the diagonal and false statements to off-diagonal elements. The red dashed line represents the performance of a random classifier.

We test our fact-checking method on tasks of increasing difficulty, and begin by considering simple factual statements in four subject areas related to entertainment, history, and geography. We evaluate statements of the form “*d*_*i*_ directed *m*_*j*_,” “*p*_*i*_ was married to *s*_*j*_,” and “*c*_*i*_ is the capital of *r*_*j*_,” where *d*_*i*_ is a director, *m*_*j*_ is a movie, *p*_*i*_ is a US president, *s*_*j*_ is the spouse of a US president, *c*_*i*_ is a city, and *r*_*j*_ is a country or US state. By considering all combinations of subjects and objects in these classes, we obtain matrices of statements (see Materials and Methods). Many of them, such as “Rome is the capital of India,” are false. Others, such as “Rome is the capital of Italy,” are true. To prevent the task from being trivially easy, we remove any edges that represent true statements in our test set from the graph. Fig 3 shows the matrices obtained by running the fact checker on the factual statements. Let *e* and *e*′ be a true and false statement, respectively, from any of the four subject areas. To show that our fact checker is able to correctly discriminate between true and false statements with high accuracy, we estimate the probability that *τ*(*e*) > *τ*(*e*′). To do so we plot the ROC curve of the classifier (see Fig 4) since the area under the ROC curve is equivalent to this probability [35]. With this method we estimate that, in the four subject areas, true statements are assigned higher truth values than false ones with probability 95%, 98%, 61%, and 95%, respectively.

### Validation on annotated corpus

**Fig 5:**
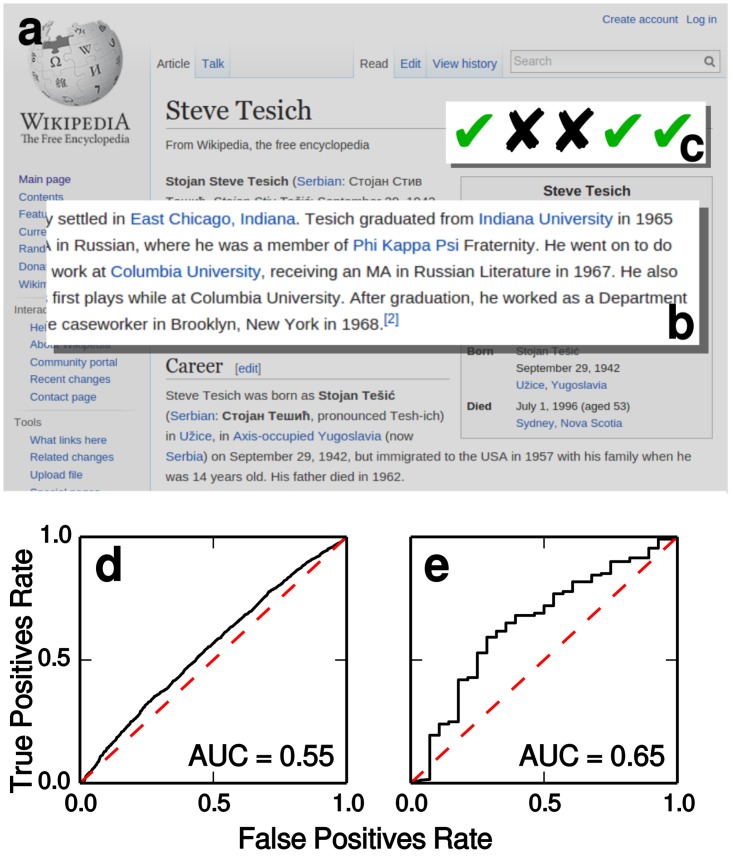
Real-world fact-checking scenario. **(a)** A document from the ground truth corpus. **(b)** Statement to fact-check: *Did Steve Tesich graduate from Indiana University, Bloomington?* This information is not present in the infobox, and thus it is not part of the WKG. **(c)** Annotations from five human raters. In this case, the majority of raters believe that the statement is true, and thus we consider it as such for classification purposes. **(d)** Receiver operating characteristic (ROC) curve of the classification for subject-predicate-object statements in which the predicate is “institution” (e.g., “Albert Einstein,” “institution,” “Institute for Advanced Studies”). A true positive rate above the false positive rate (dashed line), and correspondingly an area under the curve (AUC) above 0.5, indicate better than random performance. **(e)** ROC curve for statements with “degree” predicate (e.g., “Albert Einstein,” “degree,” “University Diploma”).

In a second task, we consider an independent corpus of novel statements extracted from the free text of Wikipedia and annotated as true or false by human raters [36] (see Materials and Methods). We compare the human ratings with the truth values provided by our automatic fact checker (Fig 5). Although the statements under examination originate from Wikipedia, they are not usually represented in the WKG, which is derived from the infoboxes only. When a statement is present in the WKG, the link is removed. The information available in the WKG about the entities involved in these particular statements is very sparse, therefore this task is more difficult than the previous case.

We find that the truth values computed by the fact checker are positively correlated to the average ratings given by the human evaluators. Table 3 shows the positive correlation between GREC human annotations and our computational truth scores.

**Table 3:** Agreement between fact checker and human raters.

Relation	*ρ*	*p*-value	*τ*	*p*-value
Degree	0.17	2 × 10^−5^	0.13	10 × 1^−6^
Institution	0.09	4 × 10^−19^	0.07	1 × 10^−24^

We use rank-order correlation coefficients (Kendall’s *τ* and Spearman’s *ρ*) to assess whether the scores are correlated to the ratings. Significance tests rule out the null hypothesis that the correlation coefficients are zero.

As shown in Fig 5, our fact checker yields consistently higher support for true statements than false ones. Using only information in the infoboxes however yields worse performance, closer to random choice: AUROC = 0.47 and 0.52 for the ‘degree’ and ‘institution’ predicates, respectively. We conclude that the fact checker is able to integrate the strength of indirect paths in the WKG, which pertain to factual information not originally included in the infoboxes.

## Discussion

These results are both encouraging and exciting: a simple shortest path computation maximizing information content can leverage an existing body of collective human knowledge to assess the truth of new statements. In other words, the important and complex human task of fact checking can be effectively reduced to a simple network analysis problem, which is easy to solve computationally. Our approach exploits implicit information from the topology of the WKG, which is different from the statements explicitly contained in the infoboxes. Indeed, if we base our assessment only on direct edges in the WKG, performance decreases significantly. This demonstrates that much of the correct measurement of the truthfulness of statements relies on indirect paths. Because there are many ways to compute shortest paths in distance graphs, or transitive closures in weighted graphs [27], there is ample room for improvement on this method.

Our WKG is built from statement of facts, which are represented as subject-predicate-object triples, i.e. information with an inherent directionality. Our results show that an undirected KG yielded the best outcomes [37, 38]. This is somehow surprising, given that in transforming a directed graph into undirected we are destroying potentially useful information. However, while some semantic relations are inherently one-way, it can be argued that some relations can be navigated in both directions (e.g. “Barack Obama” “Married-To” “Michelle Obama”) [39]. Thus we conjecture that the loss of information from disposing of the direction of edges is balanced by the possibility of finding paths, and hope that future research will elucidate this conjecture.

We live in an age of overabundant and ever-growing information, but much of it is of questionable veracity [10, 40]. Establishing the reliability of information in such circumstances is a daunting but critical challenge. Our results show that network analytics methods, in conjunction with large-scale knowledge repositories, offer an exciting new opportunity towards automatic fact-checking methods. As the importance of the Internet in our everyday lives grows, misinformation such as panic-inducing rumors, urban legends, and conspiracy theories can efficiently spread online in variety of new ways [5, 8]. Scalable computational methods, such as the one we demonstrate here, may hold the key to mitigate the societal effects of these novel forms of misinformation.

## Materials and Methods

### Wikipedia Knowledge Graph

To obtain the WKG we downloaded and parsed RDF triples data from the DBpedia project (dbpedia.org). We used three datasets of triples to build the WKG: the “Types” dataset, which contains subsumption triples of the form (subject, “is-a,” Class), where Class is a category of the DBpedia ontology; the “Properties” dataset, which contains triples extracted from infoboxes; and the DBpedia ontology, from which we used all triples with predicate “subClassOf.” This last data was used to reconstruct the full ontological hierarchy of the graph. We then discarded the predicate part of each triple and conflated all triples having the same subject and object, obtaining an edge list. In this process, we discarded all triples whose subject or object belonged to external namespaces (e.g., FOAF and schema.org). We also discarded all triples from the “Properties” dataset whose object was a date or any other kind of measurement (e.g., “Aristotle,” “birthYear,” “384 B.C.”), because by definition they never appear as subjects in other triples.

### Ideological classification of the US Congress

To get a list of ideologies we consider the “Ideology” category in the DBpedia ontology and look up in the WKG all nodes *Y* connected to it by means of a statement (*Y*, “is-a,” “Ideology”). We found *M* = 819 such nodes (see S1 Text for the complete list). Given a politician *X* and an ideology *Y* we then compute the truth value of the statement “*X* endorses ideology *Y*.” To perform the classification, we use two standard classifier algorithms: *k*-Nearest Neighbors [41] and Random Forests [42]. To assess the classification accuracy we computed F-score and area under Receiver Operating Characteristic (ROC) curve using 10-fold cross-validation.

### Simple factual statements

We formed simple statements by combining each of *N* subject entities with each of *N* object entities. We performed this procedure in four subject areas: (1) Academy Awards for Best Movie (*N* = 59), (2) US presidential couples (*N* = 17), (3) US state capitals (*N* = 48), and (4) world capitals (*N* = 187). For directors with more than one award, only the first award was used. All data were taken from Wikipedia (see S1 Text for data tables). To make the test fair, if a triple indicating a true statement was already present in the WKG, we removed it from the graph before computing the truth value. This step of the evaluation procedure is typical of link prediction algorithms [43].

### Independent corpus of statements

The second ground truth dataset is based on the Google Relation Extraction Corpus (GREC) [36]. For simplicity we focus on two types of statements, about education degrees (*N* = 602) and institutional affiliations (*N* = 10,726) of people, respectively. Each triple in the GREC comes with truth ratings by five human raters (Fig 5(c)), so we map the ratings into an ordinal scale between −5 (all raters replied ‘No’) and +5 (all raters replied ‘Yes’), and compare them to the truth values computed by the fact checker. The subject entities of several triples in the GREC appear in only a handful of links in the WKG, limiting the chances that our method can find more than one path. Therefore we select from the two datasets only triples having a subject with degree *k* > 3. Similarly to the previous task, if the statement is already present in the WKG, we remove the corresponding triple before computing the truth value.

## Supporting Information

S1 Text Data tables and list of ideologies. data tables for Fig 3 and list of ideologies used in the ideological classification of the US Congress.
(PDF)
