# Correction: Computational Fact Checking from Knowledge Networks

**DOI:** 10.1371/journal.pone.0141938

**Published:** 2015-10-27

**Authors:** Giovanni Luca Ciampaglia, Prashant Shiralkar, Luis M. Rocha, Johan Bollen, Filippo Menczer, Alessandro Flammini

There is an error in the last sentence of the “Validation on factual statements” section of the Results. The sentence should read: With this method we estimate that, in the four subject areas, true statements are assigned higher truth values than false ones with probability 95%, 98%, 100%, and 95%, respectively.

Fig 4 is incorrect. Please view the corrected figure below.

**Fig 4:**
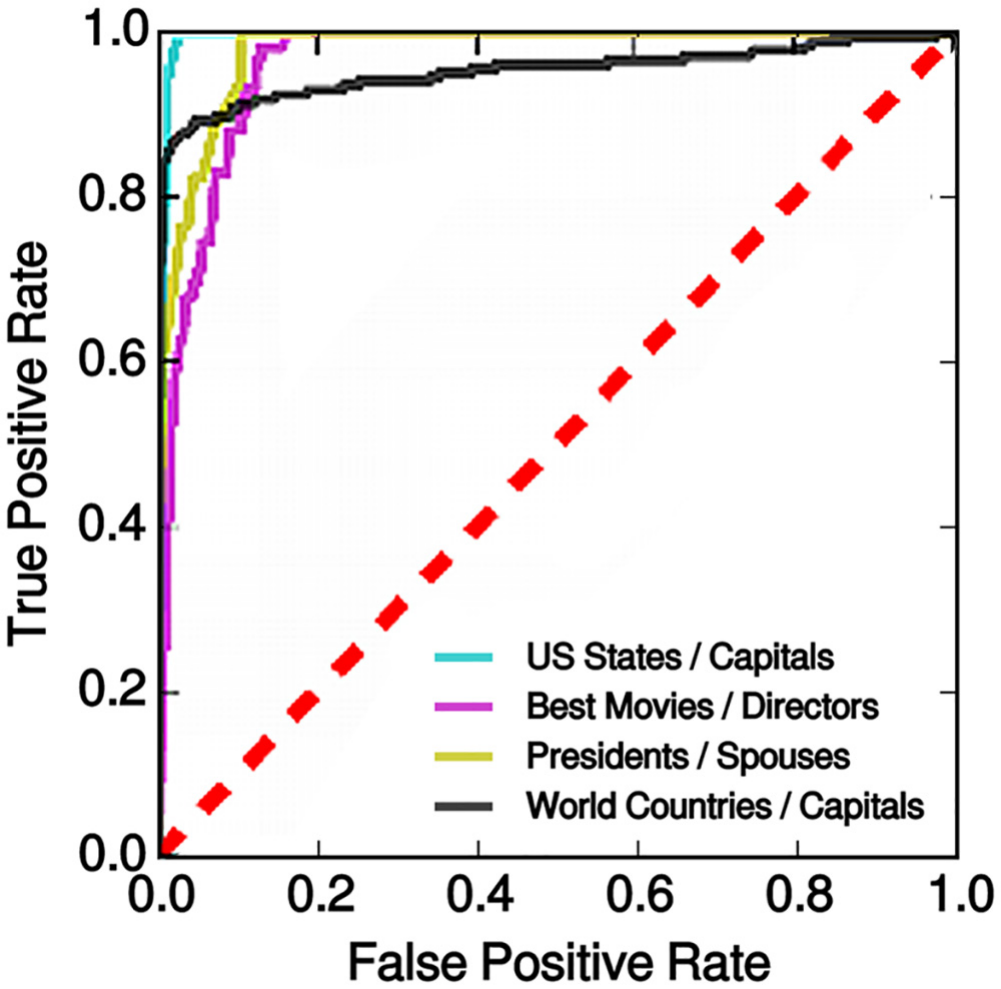
Receiver Operating Characteristic for the multiple questions task.

For each confusion matrix depicted in Fig 3 we compute ROC curves where true statements correspond to the diagonal and false statements to off-diagonal elements. The red dashed line represents the performance of a random classifier.
