# Supplementary material for: Computational Fact Checking from Knowledge Networks
Source: PLoS One. 2015 Jun 17;10(6):e0128193. doi: 10.1371/journal.pone.0128193 (PMC4471100; doi:10.1371/journal.pone.0128193)
Supplement: S1 Text — data tables for Fig 3 and list of ideologies used in the ideological classification of the US Congress. (PDF) [file pone.0128193.s001.pdf]

Computational fact checking from knowledge networks: Supporting Information

Giovanni Luca Ciampaglia,* Prashant Shiralkar, Luis M. Rocha,
Johan Bollen, Filippo Menczer, Alessandro Flammini

Data tables for Fig. 3

Table 1: Oscars for Best Movie

Year	Movie	Director
1927-28	Seventh Heaven	Frank Borzage
1927-28	Two Arabian Knights	Lewis Milestone
1928-29	The Divine Lady	Frank Lloyd
1934	It Happened One Night	Frank Capra
1935	The Informer	John Ford
1937	The Awful Truth	Leo McCarey
1939	Gone with the Wind	Victor Fleming
1942	Mrs Miniver	William Wyler
1943	Casablanca	Michael Curtiz
1945	The Lost Weekend	Billy Wilder
1948	The Treasure of the Sierra Madre	John Huston
1949	A Letter to Three Wives	Joseph L. Mankiewicz
1951	A Place in the Sun	George Stevens
1953	From Here to Eternity	Fred Zinnemann
1954	On the Waterfront	Elia Kazan
1955	Marty	Delbert Mann
1957	The Bridge on the River Kwai	David Lean
1958	Gigi	Vincente Minnelli
1964	My Fair Lady	George Cukor
1967	The Graduate	Mike Nichols
1968	Oliver	Carol Reed
1969	Midnight Cowboy	John Schlesinger
1970	Patton	Franklin J. Schaffner
1971	The French Connection	William Friedkin
1973	The Sting	George Roy Hill
1974	The Godfather Part II	Francis Ford Coppola
1976	Rocky	John G. Avildsen

*Corresponding author. Email: gciampag@indiana.edu

Table 1 – continued from previous page

Year	Movie	Director
1977	Annie Hall	Woody Allen
1978	The Deer Hunter	Michael Cimino
1979	Kramer vs Kramer	Robert Benton
1980	Ordinary People	Robert Redford
1981	Reds	Warren Beatty
1982	Gandhi	Richard Attenborough
1983	Terms of Endearment	James L. Brooks
1984	Amadeus	Miloš Forman
1985	Out of Africa	Sydney Pollack
1986	Platoon	Oliver Stone
1987	The Last Emperor	Bernardo Bertolucci
1988	Rain Man	Barry Levinson
1990	Dances with Wolves	Kevin Costner
1991	The Silence of the Lambs	Jonathan Demme
1992	Unforgiven	Clint Eastwood
1994	Forrest Gump	Robert Zemeckis
1995	Braveheart	Mel Gibson
1996	The English Patient	Anthony Minghella
1997	Titanic	James Cameron
1998	Saving Private Ryan	Steven Spielberg
1999	American Beauty	Sam Mendes
2000	Traffic	Steven Soderbergh
2001	A Beautiful Mind	Ron Howard
2002	The Pianist	Roman Polanski
2003	The Lord of the Rings The Return of the King	Peter Jackson
2005	Brokeback Mountain	Ang Lee
2006	The Departed	Martin Scorsese
2007	No Country for Old Men	Joel and Ethan Coen
2008	Slumdog Millionaire	Danny Boyle
2009	The Hurt Locker	Kathryn Bigelow
2011	The Artist	Michel Hazanavicius
2013	Gravity	Alfonso Cuarón

Table 2: Spouses of US Presidents

President		Spouse	
WW	Woodrow Wilson	EBGW	Edith Bolling Galt Wilson
WGH	Warren G. Harding	FH	Florence Harding
CC	Calvin Coolidge	GC	Grace Coolidge
HH	Herbert Hoover	LHH	Lou Henry Hoover
FDR	Franklin D. Roosevelt	ER	Eleanor Roosevelt
HST	Harry S. Truman	BT	Bess Truman
DDE	Dwight D. Eisenhower	ME	Mamie Eisenhower
JFK	John F. Kennedy	JKO	Jacqueline Kennedy Onassis
LBJ	Lyndon B. Johnson	LBJ	Lady Bird Johnson
RN	Richard Nixon	PN	Pat Nixon
GF	Gerald Ford	BF	Betty Ford
JC	Jimmy Carter	RC	Rosalynn Carter
RR	Ronald Reagan	NR	Nancy Reagan
GHWB	George H. W. Bush	BB	Barbara Bush
BC	Bill Clinton	HRC	Hillary Rodham Clinton
GWB	George W. Bush	LB	Laura Bush
BO	Barack Obama	MO	Michelle Obama

Table 3: US state capitals

Region	Capital	State
Midwest	Springfield	Illinois
	Indianapolis	Indiana
	Des Moines	Iowa
	Topeka	Kansas
	Lansing	Michigan
	Saint Paul	Minnesota
	Jefferson City	Missouri
	Lincoln	Nebraska
	Bismarck	North Dakota
	Columbus	Ohio
	Pierre	South Dakota
	Madison	Wisconsin
Northeast	Hartford	Connecticut
	Augusta	Maine
	Boston	Massachusetts
	Concord	New Hampshire
	Trenton	New Jersey
	Albany	New York
	Harrisburg	Pennsylvania
	Providence	Rhode Island
	Montpelier	Vermont
South	Montgomery	Alabama
	Little Rock	Arkansas
	Dover	Delaware
	Tallahassee	Florida
	Atlanta	Georgia U S state
	Frankfort	Kentucky
	Baton Rouge	Louisiana
	Annapolis	Maryland
	Jackson	Mississippi
	Raleigh	North Carolina
	Oklahoma City	Oklahoma
	Columbia	South Carolina
	Nashville	Tennessee
	Austin	Texas
	Richmond	Virginia
	Charleston	West Virginia
West	Juneau	Alaska
	Phoenix	Arizona
	Sacramento	California
	Honolulu	Hawaii
	Boise	Idaho
	Helena	Montana
	Carson City	Nevada
	Santa Fe	New Mexico

Table 3 – continued from previous page

Region	Capital	State
	Salem	Oregon
	Salt Lake City	Utah
	Cheyenne	Wyoming

Table 4: World country capitals

Region	Capital	State
	Angola	Luanda
	Angola	Luanda
	Benin	Porto-Novo
	Botswana	Gaborone
	Burkina Faso	Ouagadougou
	Burundi	Bujumbura
	Cameroon	Yaoundé
	Cape Verde	Praia
	Central African Republic	Bangui
	Comoros	Moroni Comoros
	Democratic Republic of the Congo	Kinshasa
	Republic of the Congo	Brazzaville
	Djibouti	Djibouti
	Egypt	Cairo
	Equatorial Guinea	Malabo
	Eritrea	Asmara
	Ethiopia	Addis Ababa
	Gabon	Libreville
	The Gambia	Banjul
	Ghana	Accra
	Guinea	Conakry
	Guinea Bissau	Bissau
	Kenya	Nairobi
	Lesotho	Maseru
	Liberia	Monrovia
	Libya	Tripoli
Africa	Madagascar	Antananarivo
	Malawi	Lilongwe
	Mali	Bamako
	Mauritania	Nouakchott
	Mauritius	Port Louis
	Morocco	Rabat
	Mozambique	Maputo
	Namibia	Windhoek
	Niger	Niamey
	Nigeria	Abuja
	Rwanda	Kigali
	São Tomé and Príncipe	São Tomé
	Senegal	Dakar
	Seychelles	Victoria Seychelles
	Sierra Leone	Freetown
	Somalia	Mogadishu
	South Africa	Pretoria
	South Sudan	Juba
	Sudan	Khartoum

Table 4 – continued from previous page

Region	Capital	State
	Swaziland	Mbabane
	Tanzania	Dodoma
	Togo	Lomé
	Tunisia	Tunis
	Uganda	Kampala
	Zambia	Lusaka
	Zimbabwe	Harare
Asia	Afghanistan	Kabul
	Armenia	Yerevan
	Azerbaijan	Baku
	Bahrain	Manama
	Bangladesh	Dhaka
	Bhutan	Thimphu
	Brunei	Bandar Seri Begawan
	Burma	Naypyidaw
	Cambodia	Phnom Penh
	China	Beijing
	Cyprus	Nicosia
	East Timor	Dili
	Georgia country	Tbilisi
	India	New Delhi
	Indonesia	Jakarta
	Iran	Tehran
	Iraq	Baghdad
	Israel	Jerusalem
	Japan	Tokyo
	Jordan	Amman
	Kazakhstan	Astana
	North Korea	Pyongyang
	South Korea	Seoul
	Kuwait	Kuwait City
	Kyrgyzstan	Bishkek
	Laos	Vientiane
	Lebanon	Beirut
	Malaysia	Kuala Lumpur
	Maldives	Malé
	Mongolia	Ulan Bator
	Nepal	Kathmandu
	Oman	Muscat Oman
	Pakistan	Islamabad
	State of Palestine	Jerusalem
	Philippines	Manila
	Qatar	Doha
	Saudi Arabia	Riyadh
	Singapore	Singapore
	Syria	Damascus

Table 4 – continued from previous page

Region	Capital	State
	Tajikistan	Dushanbe
	Thailand	Bangkok
	Turkey	Ankara
	Turkmenistan	Ashgabat
	United Arab Emirates	Abu Dhabi
	Uzbekistan	Tashkent
	Vietnam	Hanoi
Europe	Albania	Tirana
	Andorra	Andorra la Vella
	Austria	Vienna
	Belarus	Minsk
	Belgium	Brussels
	Bosnia and Herzegovina	Sarajevo
	Bulgaria	Sofia
	Croatia	Zagreb
	Czech Republic	Prague
	Denmark	Copenhagen
	Estonia	Tallinn
	Finland	Helsinki
	France	Paris
	Germany	Berlin
	Greece	Athens
	Hungary	Budapest
	Iceland	Reykjavík
	Republic of Ireland	Dublin
	Italy	Rome
	Latvia	Riga
	Liechtenstein	Vaduz
	Lithuania	Vilnius
	Luxembourg	Luxembourg
	Republic of Macedonia	Skopje
	Malta	Valletta
	Monaco	Monaco
	Montenegro	Podgorica
	Norway	Oslo
	Poland	Warsaw
	Portugal	Lisbon
	Romania	Bucharest
	Russia	Moscow
	San Marino	City of San Marino
	Serbia	Belgrade
	Slovakia	Bratislava
	Slovenia	Ljubljana
	Spain	Madrid
	Sweden	Stockholm
	Switzerland	Bern

Table 4 – continued from previous page

Region	Capital	State
	Ukraine	Kiev
	United Kingdom	London
	Vatican City	Vatican City
North America	Antigua and Barbuda	St.Johns
	Bahamas	Nassau Bahamas
	Barbados	Bridgetown
	Belize	Belmopan
	Canada	Ottawa
	Costa Rica	San José Costa Rica
	Cuba	Havana
	Dominica	Roseau
	Dominican Republic	Santo Domingo
	El Salvador	San Salvador
	Guatemala	Guatemala City
	Haiti	Port-au-Prince
	Honduras	Tegucigalpa
	Jamaica	Kingston Jamaica
	Mexico	Mexico City
	Kingdom of the Netherlands	Amsterdam
	Nicaragua	Managua
	Panama	Panama City
	Saint Kitts and Nevis	Basseterre
	Saint Lucia	Castries
	Saint Vincent and the Grenadines	Kingstown
	Trinidad and Tobago	Port of Spain
	United States	Washington D.C.
Oceania	Australia	Canberra
	Fiji	Suva
	Kiribati	South Tarawa
	Marshall Islands	Majuro
	Federated States of Micronesia	Palikir
	New Zealand	Wellington
	Papua New Guinea	Port Moresby
	Samoa	Apia
	Solomon Islands	Honiara
	Tonga	Nuku'alofa
	Tuvalu	Funafuti
	Vanuatu	Port Vila
South America	Argentina	Buenos Aires
	Bolivia	Sucre
	Brazil	Brasília
	Chile	Santiago
	Colombia	Bogotá
	Ecuador	Quito
	Guyana	Georgetown Guyana

Table 4 – continued from previous page

Region	Capital	State
	Paraguay	Asunción
	Peru	Lima
	Suriname	Paramaribo
	Uruguay	Montevideo
	Venezuela	Caracas

List of ideologies

1981 Irish hunger strike; 9/11 Truth movement; Abertzale; Abolished monarchy; African nationalism; African socialism; Afrikaner nationalism; Agorism; Agrarianism; Agrarian socialism; Ahlus Sunnah wal Jamaah (organisation); Albanians in the Republic of Macedonia; Alexander Lukashenko; Algeria; Algerianism; Alsace; Alter-globalization; American nationalism; Anarchism; Anarchist communism; Anarcho-capitalism; Anarcho-syndicalism; Andalusian nationalism; Anglo-Irish Treaty; Anglophile; Animal rights; Animal welfare; Anti; Anti-Americanism; Anti-capitalism; Anti-Catholicism in the United Kingdom; Anti-clericalism; Anti-communism; Anti Communism; Anti-corporate activism; Anti-Corruption; Anti-Esotericism; Anti-fascism; Anti-Federalism; Antifeminism; Anti-globalization movement; Anti-imperialism; Anti-Islamism; Anti-Judaism; Anti-Leninism; Anti-LGBT; Anti-liberal; Antimilitarism; Anti-nationalism; Anti-Polish sentiment; Anti-Revisionism; Antisemitism; Anti-Sovietism; Anti-Stalinist left; Anti-statism; Anti-taxation; Anti-war movement; Antiziganism; Anti-Zionism; António Ramalho Eanes; Apartheid in South Africa; Arab citizens of Israel; Arab nationalism; Arab socialism; Aragon; Armenian nationalism; Assyrian nationalism; Austrofascism; Authoritarianism; Autonomism; Awoism; Azerbaijani nationalism; Ba'athism; Balanced budget; Baloch nationalism; Bangladeshi nationalism; Basque nationalism; Bavaria; Bavarian independence; Bavarian Regionalism; Beauty; Beijing; Belarus; Bengali nationalism; Berberism; Berber people; Beta Israel; Big tent; Black Consciousness Movement; Black nationalism; Black supremacy; Bolivarianism; Bolivarian Revolution; Bolivia; Bosniaks; Bosnianism; Brahmin; Breton nationalism; Breton people; British Empire; British Fascism; British nationalism; Buddhism; Buddhist socialism; Bulgaria; Burmese Way to Socialism; Business; Caliphate; Cambodia; Canadian nationalism; Canarian nationalism; Cantonalism; Capitalism; Carinthian Slovenes; Castilian nationalism; Castroism; Catalan nationalism; Catalan separatism; Catholic Church; Catholic social teaching; Centralisation; Central Powers; Centre-left; Centre-right; Centrism; Chaldean Christians; Cham issue; Cham people (Asia); Chardal; Chavismo; Chinese nationalism; Chinese reunification; Chinese socialism; Christian communism; Christian democracy; Christian ethics; Christian humanism; Christianity; Christian left; Christian right; Christian socialism; Citizenship; Civil and political rights; Civil liberties; Classical liberalism; Classical Marxism; Clerical fascism; Clericalism; Colonialism; Communism; Communist Party of China; Communitarianism; Community politics; Conflict management; Congress of Verona (1943); Conservatism; Conservatism in Australia; Conservatism in Canada; Conservatism in Germany; Conservatism in the United States; Conservative Democrat; Conservative liberalism; Constitutionalism; Constitutional monarchy; Consumerism; Consumer protection; Cooperative; Co-operative economics; Cooperative federalism; Copyright; Cornerstone Group; Cornish Assembly; Cornish Autonomy; Cornish nationalism; Corporatism; Corsican nationalism; Cosmopolitanism; Côte d'Ivoire; Council communism; Creationism; Criticism of Islam; Croatian nationalism; Croats of Boka Kotorska; Croats of Vojvodina; Cultural conservatism; Cultural liberalism; Danish Realm; Decentralization; Decolonization; Degar; Degrowth; Demarchy; Democracy; Democratic liberalism; Democratic security; Democratic socialism; Democratic Struggle; Democratization; Deng Xiaoping Theory; Departments of Bolivia; Developmentalism; Development criticism; Devizes; Devolution; Devolved English parliament; Dictatorship of the proletariat; Direct democracy; Direct rule; Disputed status of Gibraltar; Distributism; Doi Moi; Dominant minority; Dominionism; Drug policy reform; Druze; Early Malay nationalism; Eastern Orthodox Church; Ecology; Ecology movement; Economic liberalism; Economic nationalism; Economic rationalism; Eco-socialism; Eco-sociality; E-democracy; Education; Egalitarianism; Egyptian nationalism; Éire Nua; Electoral reform; Electoral reform in New Zealand; Elitism; English independence; English nationalism; Environmentalism; Equal justice under law; Equal opportunity; Equal rights; Eritrea; Especifismo; Ethnic-minority; Ethnic nationalism; Ethnocentrism; Ethnopluralism; Eurasianism; Euro; Eurocommunism; European integration; European People's Party; Euroscepticism; Expansionism; Experimental projects; Factions in the Democratic Party (United States); Fair trade; Falangism; Family values; Far-left politics; Faroe Islands; Far right in the United Kingdom; Far-right politics; Fascism; Fathers' rights movement; Federalism; Federalism in China; Federalist; Federation;

Feminism; Feudalism; Filipino nationalism; Finland; Fiscal conservatism; Fiscal federalism; Flemish Movement; Fourth International Posadist; Francisco de Sá Carneiro; Francoist Spain; Francophile; Francophone; Franjo Tuđman; Freedom of information; Freedom of speech; Free love; Free market; Free trade; French Community of Belgium; French First Republic; French nationalism; Friesland; Frivolous political party; Fujimorism; Fundamentalism; Gaels; Gag rule; Galicianism (Galicia); Galician nationalism; Gaullism; Georgism; Gerald Götting; German Emperor; German language; German nationalism; Good governance; Goulash Communism; Grassroots; Grassroots democracy; Greater Armenia (political concept); Greater Israel; Greater Somalia; Greek nationalism; Green anarchism; Green conservatism; Greenland; Green liberalism; Green libertarianism; Green party; Green politics; Gross national happiness; Guerrilla warfare; Guevarism; Gwynedd; Halakha; Haredi Judaism; Harm reduction; Hazara people; Hindutva; Ho Chi Minh Thought; Holism; Honduras; House of Grimaldi; Hoxhaism; Hugo Chávez; Humanism; Humanist Movement; Human rights; Hungarian nationalism; Hungarians in Romania; Hungarians in Slovakia; Husakism; Hutu Power; Idealism; Imperial Preference; Impossibilism; Independence; Independent (politician); Indian nationalism; Indigenism; Indigenous rights; Individualism; Industrialisation; Integral humanism; Integralism; Intellectual property; Internal resistance to South African apartheid; Internationalism; Internationalism (politics); International Socialist Tendency; Internet censorship; Iranian nationalism; Iranian reform movement; Iraqi nationalism; Iraqi Turkmens; Irish nationalism; Irish republicanism; Iron Guard; Islam; Islamic democracy; Islamic fundamentalism; Islamic republic; Islamism; Islamophobia; Isolationism; Israeli–Palestinian conflict; Istria; Italian nationalism; Italy–Malta relations; Ivoirité; Jadid; James Madison; Japanese militarism; Japanese nationalism; J. B. Danquah; Jeffersonian democracy; Juche; Justice; Justicialist Party; Kahanism; Katarismo; Kemalist ideology; Ketuanan Melayu; Keynesian economics; Kham; Khatim an-Nabuwwah; Khmer people; Kidderminster; Kirchnerism; Korean nationalism; Kuomintang; Kurdish nationalism; Kurdish people; Kwame Nkrumah; Kyrgyz nationalism; Laborer; Labor rights; Labor Zionism; Labour movement; Laïcité; Laissez-faire; Land of Israel; Latvia; Latvian people; Law and order (politics); Lebanese nationalism; Left centrism; Left communism; Left-libertarianism; Left–right politics; Left-wing nationalism; Left-wing politics; Legality of cannabis; Leninism; Liberal conservatism; Liberal democracy; Liberalism; Liberalism in Australia; Liberalism in Colombia; Liberalism in South Korea; Liberalism in the United States; Liberal movements within Islam; Liberal nationalism; Liberal socialism; Liberation theology; Liberism; Libertarian conservatism; Libertarian Democrat; Libertarianism; Libertarian Marxism; Libertarian socialism; Lieberman Plan; Lists of active separatist movements; Lithuania; Localism; Localism (politics); Luxemburgism; Macedonian nationalism; Malta–United Kingdom relations; Maltese; Maoism; Market liberalism; Market socialism; Martinique; Marxism; Marxism–Leninism; Masculism; Mass politics; Megali Idea; Metaxism; Microeconomic reform; Militant; Militarism; Millî Görüş; Minarchism; Minority rights; Miraism; Mixed economy; Mizrahi Jews; Mobutism; Moderate; Moderate Islamism; Moderation; Modernization; Modern Orthodox Judaism; Monarchism; Monarchy; Monarchy of Australia; Monetary reform; Montenegrin nationalism; Morality; Moravism; Morocco; Movement for the unification of Romania and Moldova; Multiculturalism; Multiethnic society; Muslim; Nacionalismo (Argentine political movement); Naga Nationalism; Nasserism; National Bolshevism; National Catholicism; National communism; National conservatism; Nationalism; Nationalization; National liberalism; National Liberation (historical); National Reconciliation; National syndicalism; Nativism (politics); Natural Capitalism: Creating the Next Industrial Revolution; Nazism; Neocolonialism; Neoconservatism; Neoconservatism (disambiguation); Neo-fascism; Neoliberalism; Neo-Nazism; Netherlands; Network neutrality; Neutrality (international relations); Nevis; New Democrats; New Left; New Nationalism; New Right; None of the above; Non-interventionism; Nonpartisan; Nonsectarian; Nonviolence; Nonviolent resistance; Non-voting; Nordic agrarian parties; Norse religion; Norwegian romantic nationalism; Objectivism (Ayn Rand); One country, two systems; One nation conservatism; Open government; Opposition (politics); Opposition to immigration; Oromo people; Pacifism; Pakistani nationalism; Paleoconservatism; Paleolibertarianism; Palestinian nationalism; Palestinian sovereignty; Pan-Africanism;

Panama; Pan-Americanism; Pan-Arabism; Pañcasīla; Pancasila (politics); Pan-European identity; Pan-European nationalism; Pan-Germanism; Pan-Iranism; Pan-Islamic awakening; Pan-Islamism; Pan-Latin Americanism; Pan-Slavism; Pan-Turkism; Paraguay; Parliamentary system; Participatory democracy; Participatory politics; Partition of Belgium; Pashtun people; Patent; Patriotism; Peace; Peace movement; Pensioner; Pensioners' Party; People of Ethiopia; Peronism; Personalism; Peru; Peter Kropotkin; Pim Fortuyn; Pim Fortuyn List; Platformism; Pochvennichestvo; Poles in Lithuania; Political corruption; Political freedom; Political parties of minorities; Political positions of David Cameron; Political radicalism; Political satire; Politics of Israel; Popolarismo; Popular front; Popular Socialism; Populism; Portuguese nationalism; Portuguese people; Pragmatism; Presentation program; President of the United States; President of Ukraine; Privacy; Pro-Europeanism; Progressive Christianity; Progressive Democrats of America; Progressivism; Progressivism in the United States; Protectionism; Protest; Protestantism; Protest vote; Proto-fascism; Proxy voting; Publicly funded health care; Puerto Rican independence movement; Quebec sovereignty movement; Qutbism; Racialism; Radical; Radicalism (historical); Rakhine people; Rankovićism; Redistribution of wealth; Reform; Reformism; Reform movement; Regional development; Regionalism (politics); Regions of Ethiopia; Reintegrationism; Religion; Religious denomination; Religious nationalism; Religious Zionism; Republicanism; Republicanism in Australia; Republicanism in New Zealand; Republicanism in the United Kingdom; Republicanism in the United States; Revisionism (Marxism); Revisionist Zionism; Revolutionary socialism; Rhodesia; Right-libertarianism; Rights; Right-wing politics; Right-wing populism; Royalist; Ruhollah Khomeini; Rule of law; Russia; Russian immigration to Israel in the 1990s; Russian nationalism; Russians in Estonia; Russians in Latvia; Russians in Lithuania; Russo-centrism; Russophilia; Sahrawi people; Salafi; Sami people; Sandinismo; Satire; Savoy; Scientific development concept; Scientific socialism; Scottish independence; Scottish Labour Party; Scottish national identity; Scottish nationalism; Secluarism; Secular humanism; Secularism; Secularism in Pakistan; Secularity; Self-determination; Senior citizen; Separatism; Sephardic Haredim; Serbia; Serbian–Montenegrin unionism; Serbian nationalism; Serbian progressivism; Serbs of Montenegro; Sex-positive movement; Shia Islam; Sindhi nationalism; Single-issue politics; Sinhalese Buddhist nationalism; Slavic nationalism; Slavonia; Slovaks; Slovenian nationalism; Small government; Social change; Social conservatism; Social Conservatism; Social conservatism in the United States; Social corporatism; Social Credit; Social democracy; Social ecology; Social humanism; Social Individualism; Socialism; Socialism and Islam; Socialism of the 21st century; Socialist economics; Socialist feminism; Social Justce; Social justice; Social liberalism; Social market economy; Social republicanism; Social sphere; Solidarism; Somalia; Songun; South Africa; South Sudan; Souverainism; Sovereignty; Spain; Spanish nationalism; Spiritualism; Spirituality; Sri Lankan Tamil nationalism; Stalinism; State Peace and Development Council; State Shinto; States' rights; Statism; Statism in Shōwa Japan; Strasserism; Structuralism; Sudan; Šumadija; Sunni Islam; Sunshine Policy; Sustainable development; Sweden; Swedish-speaking Finns; Syncretic politics; Syndicalism; Syrian nationalism; Syrmia; Szeged Idea; Taiwanese nationalism; Taiwan independence; Taiwanization; Tamil nationalism; Technocracy; Temperance movement; Tertium quids; Thatcherism; Third camp; Third Position; Third Way; Third Way (centrism); Third Way (United Kingdom); Three Principles of the People; Three Represents; Tigray-Tigrinya people; Titoism; Torah; Trade union; Traditionalism; Traditionalist conservatism; Transcendental Meditation; Transnistria; Transparency (behavior); Treaty of Lisbon; Tribalism; Triple Entente; Tripuri nationalism; Trotskyism; Truth and reconciliation commission; Turkic nationalism; Turkish nationalism; Two-Nation Theory; Two-state-solution; Ujamaa; Ukrainian nationalism; Ulster loyalism; Ulster nationalism; Ultramontanism; Unification Church; Unilateral Declaration of Independence; Unionism in Ireland; Unionism in Scotland; Unionism in the United Kingdom; Union of European Federalists; Union State; Unitarisation; United Ireland; United States Congress; Urban design; Uribism; Uruguay; Uzbekistan; Valencian nationalism; Venetian nationalism; Venizelism; Vietnam; Vojvodina; Völkisch movement; Volksgemeinschaft; Voluntaryism; Walloon Movement; Warsaw; Wars of national liberation; Weapons Rights; Welfare; Welfare State; Welsh independence; Welsh nationalism; Wessex; Western conservatism; Western

world; Whiggism; White nationalism; White separatism; White supremacy; Solidarity; Women's rights; Workerism; Xenophobia; Yalkut Yosef; Youth rights; Zionism; Zulu people.
